# Exposure to urban PM1 in rats: development of bronchial inflammation and airway hyperresponsiveness

**DOI:** 10.1186/s12931-016-0332-9

**Published:** 2016-03-10

**Authors:** Ágnes Filep, Gergely H. Fodor, Fruzsina Kun-Szabó, László Tiszlavicz, Zsolt Rázga, Gábor Bozsó, Zoltán Bozóki, Gábor Szabó, Ferenc Peták

**Affiliations:** Department: MTA-SZTE Research Group on Photoacoustic Spectroscopy, H-6720 Szeged, Dóm tér 9 Hungary; Department of Optics and Quantum Electronics, University of Szeged, H-6720 Szeged, Dóm tér 9 Hungary; Department of Medical Physics and Informatics, University of Szeged, H-6720 Szeged, Korányi fasor 9 Hungary; Institute for Environmental Sciences, University of Szeged, H-6720 Szeged, Dóm tér 9 Hungary; Department of Pathology, University of Szeged, H-6720 Szeged, Állomás u. 2 Hungary; Department of Mineralogy, Geochemistry and Petrology, University of Szeged, H-6722 Szeged, Egyetem u. 2 Hungary

**Keywords:** Air pollution, Lung function, Airway hyperresponsiveness, Bronchial inflammation, Ambient aerosol

## Abstract

**Background:**

Several epidemiological and laboratory studies have evidenced the fact that atmospheric particulate matter (PM) increases the risk of respiratory morbidity. It is well known that the smallest fraction of PM (PM1 - particulate matter having a diameter below 1 μm) penetrates the deepest into the airways. The ratio of the different size fractions in PM is highly variable, but in industrial areas PM1 can be significant. Despite these facts, the health effects of PM1 have been poorly investigated and air quality standards are based on PM10 and PM2.5 (PM having diameters below 10 μm and 2.5 μm, respectively) concentrations. Therefore, this study aimed at determining whether exposure to ambient PM1 at a near alert threshold level for PM10 has respiratory consequences in rats.

**Methods:**

Rats were either exposed for 6 weeks to 100 μg/m^3^ (alert threshold level for PM10 in Hungary) urban submicron aerosol, or were kept in room air. End-expiratory lung volume, airway resistance (R_aw_) and respiratory tissue mechanics were measured. Respiratory mechanics were measured under baseline conditions and following intravenous methacholine challenges to characterize the development of airway hyperresponsiveness (AH). Bronchoalveolar lavage fluid (BALF) was analyzed and lung histology was performed.

**Results:**

No significant differences were detected in lung volume and mechanical parameters at baseline. However, the exposed rats exhibited significantly greater MCh-induced responses in R_aw_, demonstrating the progression of AH. The associated bronchial inflammation was evidenced by the accumulation of inflammatory cells in BALF and by lung histology.

**Conclusions:**

Our findings suggest that exposure to concentrated ambient PM1 (mass concentration at the threshold level for PM10) leads to the development of mild respiratory symptoms in healthy adult rats, which may suggest a need for the reconsideration of threshold limits for airborne PM1.

## Background

Epidemiologic studies have observed associations between short-term increases in ambient particulate matter (PM) concentrations and increases in respiratory morbidity [1]. Atmospheric aerosol is a complex mixture of gases, solid and liquid particles. The diameter of these particles (Dp) varies in five orders of magnitude (1 nm – 100 μm). It has been well established that the particle size significantly determines how deep the particles can penetrate into the lung compartments. Particles with diameters between 2.5 and 10 μm (usually defined as PM2.5 and PM10) deposit mainly in the upper airways and can be cleared by the mucociliary system. PM2.5 deposit in the tracheobronchial region, whereas PM1 (particles with diameters of less than 1 μm) can reach the lung periphery, i.e. the alveolar region [2]. Although in urban PM mass PM10 is dominant, in industrial areas the PM1/PM10 mass ratio can exceed 0.5 [3]. Such a high mass ratio expressed in particle number (i.e. nPM1/nPM10) means at least 3 orders of magnitude. Several studies have demonstrated that low emission zones (LEZ) have a far greater positive effect on public health than one would expect from PM10 data [4]. The reason for this benefit is that LEZ are effective in decreasing the number of small particles, but not the mass concentration of any size fraction of PM. Because of these evidences, in the last decade the scientific interest has shifted from PM10 and PM2.5 to PM1 [5], even though air quality standards related to PM1 are still nonexistent.

The chemical composition of PM particles and their adverse health effects vary greatly according to their emission sources. The pulmonary effects of specific, potentially harmful constituents of PM, such as iron [6], elemental carbon [7] or combustion-derived nanoparticles [8] have been investigated. However, it is questionable whether these findings can be generalized to humans exposed to PM because of the complexity of real atmospheric aerosol [9]. The few earlier studies assessing the respiratory consequences of complex atmospheric aerosols in animal models were limited to exposures to particle concentrations at least five times higher than the alert level [10–13]. Thus, the development of adverse pulmonary symptoms including bronchial inflammation and airway hyperresponsiveness could be anticipated [14]. Consequently, it is not known whether complex urban aerosols in the PM1 fraction with concentrations around the current threshold level for PM10 cause pulmonary symptoms in healthy adult individuals. Therefore, the present study aims to establish whether the prolonged (6 weeks) inhalation of urban PM1 in concentrations at the current alert PM10-related threshold level has pulmonary effects on healthy adult rats.

## Methods

Ethical approval for this study (no. I-74-50/2012) was provided by the Experimental Ethics Committee of the University of Szeged, Szeged, Hungary (Chairperson Prof. Gy. Szabó) on 7 December 2012, and by the local office of the Hungarian Animal Health and Welfare Directorate (no. XIV/152/2013, Chairperson Cs. Farle) on 9 January 2013. The work was carried out in accordance with EU Directive 2010/63/EU relating to animal experiments.

### Exposure to PM1

Atmospheric aerosol samples were collected for a period of 5 years continuously in the Combined Cycle Power Plant of Debrecen, the second largest city in Hungary. The filtration system of the power plant operates 5,000 h per year and extracts approximately 580,000 m^3^ air in an hour. Particle removal is achieved in three steps. As many as 180 pieces of coarse filters are responsible for the removal of particles above 63 μm, and the same number of glass fiber filters for the removal of particles between 63 and 1 μm. The remaining small particles are removed from the air of the turbine areas by washing with water. The filtration properties of the filters depend strongly on the actual filter loading. In the initial phase, particles are caught between the fibers, but as the filter becomes more loaded, particles deposit on the top of the filter. As the particles occlude the routes within the filter, the position of the deposition efficiency minimum on the particle size axis shifts towards the smaller sizes. A complete characterization of the sample can be found in a previous study, which demonstrated that 88 % of the particles collected from the coarse filters were below 63 μm [14]. In the present study we used particles collected from the glass fiber filters. To be able to collect submicron particles without any extraction we aspirated the particles from the surface of the glass fiber filters with a special hoover. Additional size selection was done during the resuspension process.

**Fig. 1:**
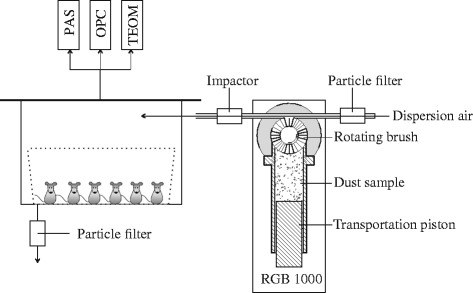
Experimental setup for PM1 exposure. PAS: photoacoustic spectrometer, OPC: optical particle counter, TEOM: tapered element oscillation microbalance

The main air pollution sources at the sampling point were associated with the busy roads nearby, a residential area and the central railway station. Because of the long sampling period, dust composition can be interpreted as typical urban PM in any Central European city [15]. In order to achieve a more physiological deposition, we opted to choose aerosolized particle exposition, rather than intratracheal instillation. The aim of our study was to approach the ambient exposure as much as possible; hence we selected whole body exposure. The PM1 test atmosphere was created inside an exposure chamber. The total volume of the exposure chamber was 60 l, and the animal load (i.e. the total body volume of the animals) at the end of the experiment was 3.8 %. That ratio meets Silver’s recommendation [16] to minimize effects on exposure concentration related to animal surface area. Re-suspension of the dust was achieved by using a PALAS RGB1000 disperser (Fig. 1) with a Type C dispersion cover (7 mm diameter powder reservoir), which uses a rotating brush to channel the particles into the dispersion airflow. The characteristics of the aerosol inside the chamber were evaluated at multiple points of the chamber before the study and continuously monitored during the 6-week-long exposure procedure. The mass concentration of the generated aerosol (ρ) was measured with a tapered element oscillating microbalance (TEOM) instrument (Series 1400a, Rupprecht and Patashnick Co. Inc., Albany, NY, USA) and particle number size distribution (dN/dlogDp) with an optical particle counter (OPC, Model 1.109, Grimm Aerosol Technik, Ainring, Germany). Black carbon content was also continuously measured by a photoacoustic spectroscopy (PAS) based instrument (courtesy of Hilase Ltd.). The characteristics of the achieved atmosphere were controlled by the settings of the disperser (feed rate of the transportation piston holding the particle sample, speed of the rotating brush and flowrate of dispersion air) and by using an in-house developed PM1 impactor in front of the exposure chamber. The cut-off diameter of the impactor at the applied flowrate was previously modeled and bench tested. In our case the flowrate of dispersion air was set to 8.3 l/min, which satisfied both the needs of the animals and the connected instruments (sample flow of TEOM, OPC and PAS was 3, 1.5 and 1.5 l/min, respectively). The feed rate was 20 mm/h and the speed of the brush rotation was 600 min^−1^. The applied flowrate ensured more than eight total volume changes per hour. The applied maximum animal load (3.8 %) and eight volume changes per hour lead to less than 3 ppm ammonia concentration in the chamber at the end of an exposure period (6 h) according to Dorato and Wolf [17]. Atmospheric pressure in the chamber was maintained through an open line (via a disposable particle filter, in order to avoid contamination of the air in the room). Relative humidity in the chamber was controlled by using zeolite.

Two groups of male Wistar rats were studied (weight range 350–455 g, 380 ± 36 g in the exposed group and 405 ± 27 g in the control group). The animals were maintained at a 12 h day/night cycle. The animals in the exposed group were exposed to PM1 in the exposure chamber for 6 h a day (09:00–17:00), 5 days a week, for 6 weeks (*n* = 6). The animals in the control group were kept in another chamber with identical dimensions. They underwent the same procedure except that they were allowed to breathe particle-free room air (*n* = 6). The rats in both groups had access to food and water ad libitum throughout the entire exposure period. Both groups were examined following this 6-week-long exposure.

### PM1 mass concentration

To verify the stability of the target 100 μg/m^3^ mass concentration of PM1 inside the chamber, a TEOM was used. This instrument allows the quasi-continuous monitoring of the mass of PM accumulating on a filter mounted on an oscillating microbalance inside the measurement apparatus [18]. Changes in the frequency of oscillation, which reflect the mass of material accumulating on the filter, are detected in quasi-realtime and are converted by a microprocessor into an equivalent PM mass concentration every few seconds with a 10 min running average. The TEOM air stream was heated to 40 °C to prevent the condensation of water vapor on the collected samples and to keep the non-water semi-volatile mass loss at minimum [19].

### PM1 particle number size distribution

OPC that was used for the real-time characterization of the particle number size distribution [20] detects light scattering on an individual particle passing through a laser beam. This device uses a 683 nm laser diode to illuminate the beam containing the particles, and a wide-angle collector optic is used to detect the subsequent light pulses with a photodiode. By knowing the geometry and flow parameters, the optical diameter, the size distribution and the total concentration of particles can be calculated from the intensity of scattered light.

### Chemical composition of PM1

The elemental composition of the sample was measured with a RIGAKU Supermini WD-XRF (Pd X-ray source, 50 kV excitation voltage, 40 mA anode current) based on the emission of characteristic “secondary” (or fluorescent) X-rays (XRF) from a material that has been excited through bombardment with high-energy X-rays or gamma rays. Even though XRF is one of the most reliable methods for elemental composition measurement, quantification of the carbon content is not possible. Therefore, we measured the total carbon (TC) and the black carbon (BC) content of the aerosol separately.

The total carbon (TC) content of the sample was measured with the catalytic oxidation method (Elementar Analysensysteme GmbH), which achieves total combustion of samples by heating them to 1200 °C in an oxygen-rich environment inside the TC combustion tubes filled with a platinum catalyst. The carbon dioxide generated by oxidation was detected using a nondispersive infrared sensor (NDIR).

The black carbon (BC) content of PM1 was measured real-time with a photoacoustic spectroscopy (PAS) based instrument (courtesy of Hilase Ltd.) using a 680 nm laser diode. This method is based on the formation of sound waves following light absorption in a material sample [21]. PAS is the only method that is able to detect the optical absorption of particles in their natural airborne state.

### Lung volume measurements

End-expiratory lung volume (EELV) was measured in both groups by using a body plethysmograph as detailed earlier [22], following tracheostomy but preceding vessel preparations. Briefly, the trachea was occluded at end-expiration until 3 or 4 spontaneous inspiratory efforts had been generated by the animal in the closed box. The changes in tracheal pressure and plethysmograph box pressure during these maneuvers were recorded, and Boyle’s law was applied to calculate EELV from the relationship between the tracheal pressure and the box pressure after correction for the box impedance [23]. To minimize the biasing effects of the different breathing frequencies during the inspiratory efforts, the box pressure data were corrected for the thermal characteristics of the plethysmograph.

### Measurement of airway and respiratory tissue mechanics

The input impedance of the respiratory system (Z_rs_) was measured by applying the forced oscillation technique in short (6 s) end-expiratory pauses interposed in the mechanical ventilation, as detailed previously [24]. Briefly, the ventilation was stopped at end-expiration and the tracheal cannula was connected to a loudspeaker-in-box system instead of the ventilator circuit, delivering a computer-generated small-amplitude (<1 cmH_2_O) pseudorandom signal (23 non-integer multiples between 0.5 and 20.75 Hz) through a 100 cm long, 2 mm internal diameter polyethylene tube into the tracheal cannula. Lateral pressures were measured by using two identical pressure transducers (model 33NA002D, ICSensors, Milpitas, CA, USA) at the loudspeaker end (P_1_) and at the tracheal end (P_2_) of the wave-tube. The signals P_1_ and P_2_ were low-pass filtered (5th order Butterworth, 25 Hz corner frequency), and sampled with the analogue-digital board of a microcomputer at a rate of 256 Hz. Fast Fourier transformation with 4 s time windows and 95 % overlapping was used to assess the pressure transfer functions (P_1_/P_2_) from the 6 s recordings collected during apnoea. Zrs was calculated as the load impedance of the wave-tube using Eq. 1 [25]:

1 $$ {Z}_{rs}=\frac{Z_0\cdot \sinh \kern0.4em \left(\gamma L\right)}{\frac{P_1}{P_2}- \cosh \kern0.4em \left(\gamma L\right)} $$

where Z_o_ is the characteristic impedance and γ is the complex propagation wave number. These parameters were determined based on the geometrical data and the material constants of the wave-tube and the air.

The input impedances of the tracheal cannula and the connections were also measured, and subtracted from each Z_rs_ spectrum.

A model described by Eq. 2, containing a frequency-independent resistance (R_aw_) and inertance (I_aw_) and a tissue damping (G) and elastance (H) of a constant-phase tissue compartment [26] was fitted to the Z_rs_ spectra by minimizing the weighted difference between the measured and the modelled impedance data.

2 $$ {Z}_{rs}={R}_{aw}+j\cdot \omega \cdot {I}_{aw}+\frac{G-j\cdot H}{\omega^a} $$

where α is equal to (2/π)atan(H/G), ω is the angular frequency and j is the imaginary unit.

The tissue parameters G and H are attributed to the damping (resistive) and elastic properties of the respiratory system. R_aw_ and I_aw_ represent primarily the resistance and inertance of the airways, since the contribution of the chest wall to these parameters in rats is minor [27].

### Animal preparations

Anesthesia was induced with an intraperitoneal injection of sodium pentobarbital (45 mg/kg) in adult male Wistar rats (393.3 g, 340–450 g). A polyethylene cannula (16 gauge, B. Braun Melsungen AG, Melsungen, Germany) was initiated through tracheostomy after subcutaneous administration of local anasthetics (lidocaine, 2–4 mg/kg) to ensure adequate analgesia around the surgical wound. The rats were then placed on a heating pad in a supine position with the tracheal tube connected to a small animal ventilator (Model 683, Harvard Apparatus, South Natick, MA, USA), to allow mechanical ventilation with room air (70 breaths/min, tidal volume 7 ml/kg). Then a femoral vein was cannulated (Abocath 22 G) for drug delivery. Anesthesia was also maintained through this iv line by regular injections of sodium pentobartibal (12 mg/kg, every 30 min). A femoral artery was also catheterized (Abocath 22 G) and attached to a pressure transducer (Model TSD104A, Biopac, Santa Barbara, CA, USA) for continuous systemic blood pressure monitoring to assess mean arterial pressure. The arterial blood pressure, ECG and heart rate were monitored continuously with a data collection and acquisition system (Biopac, Santa Barbara, CA, USA). Body temperature was kept in the 37 ± 0.5 °C range by using the heating pad. Muscle relaxation was achieved by repeated iv administration of pipecuronium (0.1 mg/kg, every 30 min, Arduan, Richter-Gedeon, Budapest, Hungary).

### Experimental protocol

Both groups underwent the same experimental procedure. Following the tracheostomy the animals were placed in the plethysmograph box, and 3 to 4 EELV recordings were performed as detailed above. Mechanical ventilation was then maintained during the surgical preparations. After the animals had reached a steady-state condition, the volume history was standardized by performing lung hyperinflation by occluding the expiratory port of the ventilator. Baseline (BL) respiratory mechanical properties were determined by measuring 3 to 4 reproducible Z_rs_ data sets. To assess the appearance of airway hyperresponsiveness subsequent to the exposures, continuous iv infusions of methacholine (MCh) were administered with increasing doses (4, 8 and 16 μg/kg/min). A set of Z_rs_ data including 3 to 4 recordings was recorded 5 min after the onset of the infusion at each dose. Following the last dose, MCh infusion was stopped and after a 30 min recovery period, another set of Z_rs_ data was collected as previously. At the end of the protocol, bronchoalveolar lavage was performed on the left lung, as detailed below. The right lung was fixed and excised for histological analyses.

### Bronchoalveolar lavage

To assess pulmonary inflammatory cell counts, bronchoalveolar lavage of the left lung was performed. Following the euthanasia of the animals with an overdose of sodium pentobarbital, a mid-line thoracotomy was performed and the right bronchus was localized and clamped. Then 4 ml of pre-warmed (37 °C) normal saline was injected into the tracheal tube and the animal was re-connected to the ventilator for 1 min and the bronchoalveolar lavage fluid (BALF) was suctioned. Following the suctioning the clamp on the right bronchus was released. The samples were centrifuged onto a slide using a cytocentrifuge and following overnight drying, they were stained with haematoxylin-eosin and manually counted under a light microscope from 20 randomly selected non-overlapping fields of vision. The average number of specific cell types and the average total cell count were calculated.

### Lung histopathological examinations

The right lungs, which had not been lavaged previously, were used for these analyses. The lungs were filled with 4 % buffered formalin by applying a hydrostatic pressure of 20 cmH_2_O. The lungs and heart were then removed *en bloc* and placed into 4 % buffered formalin until processing.

#### Light microscopy

After complete fixation, transhilar horizontal sections (perpendicular to the longitudinal axis of the lung from the hilum) were embedded in paraffin. Two 5 μm sections were prepared in each lung specimen and were stained with haematoxylin-eosin.

#### Electron microscopy

For transmission electron microscopy, the formalin fixed, paraffin embedded specimens were re-embedded into plastic (Embed812, EMS, USA), and 70 nm thick sections were cut and placed on oval slot copper grids. They were analyzed under a transmission electron microscope (Philips CM10, 100 KV).

### Statistical analyses

The scatters in the parameters were expressed as SE values. The Kolmogorov-Smirnov test was used to test data for normality. Two-way repeated measures of analysis of variances (ANOVA) with the factors assessment time and group allocation were used to assess the effects of fine particles on the respiratory mechanical parameters. The Holm-Sidak multiple comparison procedure was applied to compare the different experimental conditions (for repeated measures) or groups (for independent groups). Differences of EELV, baseline mechanical parameters and BALF cell counts were detected by Student’s t-test. Statistical tests were carried out with the SigmaPlot software package (version 12.5, Systat Software, Inc., CA, USA) with a significance level of *p* < 0.05.

## Results

**Fig. 2:**
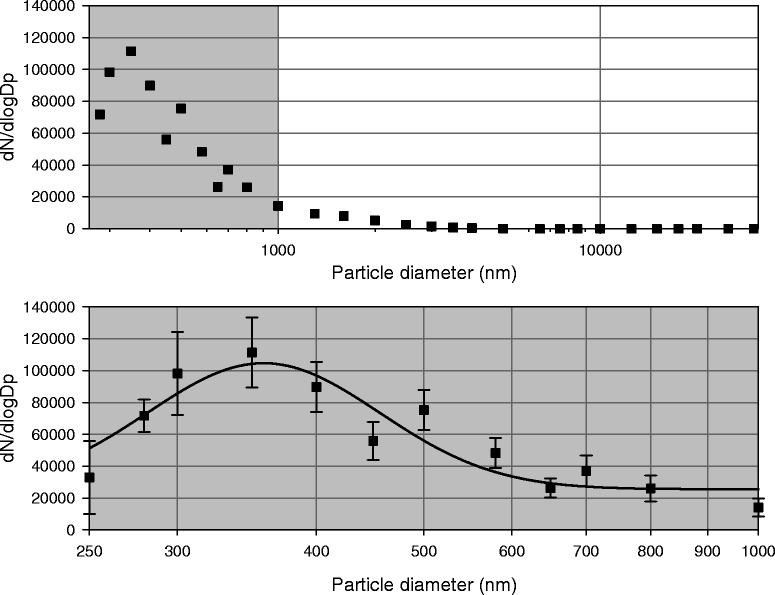
Average particle number size distribution in the exposure chamber. The *top* panel shows measured values in the whole range, the *bottom* panel is a zoomed view of the gray area in the *top* panel. Symbols are average values with SD, continuous line represents the calculated Gaussian fit

The average PM1 concentration during the exposure periods was 101.7 ± 29.4 μg/m^3^. The particle number size distribution in the exposure chamber was unimodal. The geometric mean diameter was calculated by Gaussian fit, and was found to be 391.2 ± 21.3 nm (Fig. 2). The geometric mean diameter based on particle mass size distribution (assuming a constant density) was found to be 2859.8 ± 139.7 nm. The fact that the ratio of particles having diameter larger than 1 μm was 4.87 % (in number concentration) clearly shows that particle mass size distribution can be misleading in case of dominating small particles.

The analysis of the chemical composition of the PM1 samples revealed the predominance of carbon (TC = 33.4 % containing BC = 6.38 %). Among the remaining chemical elements, silica was present in the greatest quantity (Si = 17.6 %), followed by iron (Fe = 11.4 %), calcium (Ca = 8.46 %) and aluminum (Al = 5.12 %). Lesser, but still noticeable amounts of sulfur (S = 2.32 %) and chlorine (Cl = 1.9 %) were found. Other metals were present in the samples in trace amounts (Ti = 0.67 %, Cu = 0.14 %, Zn = 0.29 %, Pb = 0.07 %).

There was no detectable difference between the two groups in terms of body weight (*p* = 0.235). The baseline values of EELV and respiratory mechanical parameters are displayed in Table 1. No statistically significant difference was detected between the control and exposed groups in any of these parameters.

**Table 1:** Baseline values of end-expiratory lung volume (EELV) and respiratory mechanical parameters (airway resistance, R_aw_; tissue damping, G and tissue elastance, H)

	EELV (ml/kg)	R_aw_ (cmH_2_O.s/l)	G (cmH_2_O/l)	H (cmH_2_O/l)
Control Group	11.2 ± 0.5	48.0 ± 2.4	955.7 ± 26.2	3750.0 ± 140.6
Exposed Group	11.7 ± 0.5	47.7 ± 2.7	952.3 ± 51.1	3643.5 ± 179.5

**Fig. 3:**
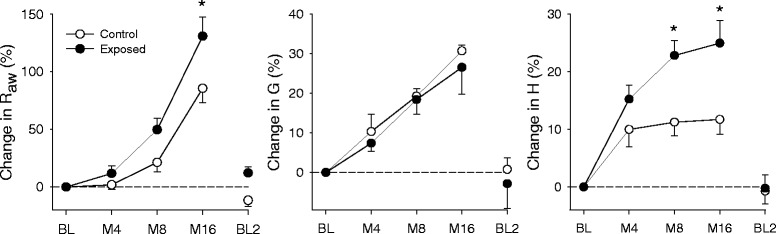
Changes of the respiratory mechanical parameters following methacholine challenge. R_aw_: airway resistance, G: tissue damping, H: tissue elastance, BL: baseline, M4-8-16: methacholine doses of 4-8-16 μg/kg/min. *: *p* < 0.05 vs. control group

Figure 3 depicts the effects of MCh provocation on the respiratory mechanical parameters. All parameters exhibited elevations relative to the baseline in a dose-dependent manner. However, the animals in the exposed group exhibited significantly greater responses to 8 μg/kg/min MCh in H (*p* = 0.011), and to 16 μg/kg/min MCh in R_aw_ (*p* = 0.005) and H (*p* = 0.006). MCh-induced changes in G did not differ between the groups throughout the study. All parameters returned to their baseline values after the 30 min recovery period (BL2).

For the parameter R_aw_ provocative dose (PD_50-Raw_) was calculated via linear interpolation, representing the dose of MCh associated with a 50 % increase in R_aw_. PD_50-Raw_ was significantly lower in the exposed group (4.299 ± 0.509 μg/kg/min vs. 5.88 ± 0.513 μg/kg/min).

**Fig. 4:**
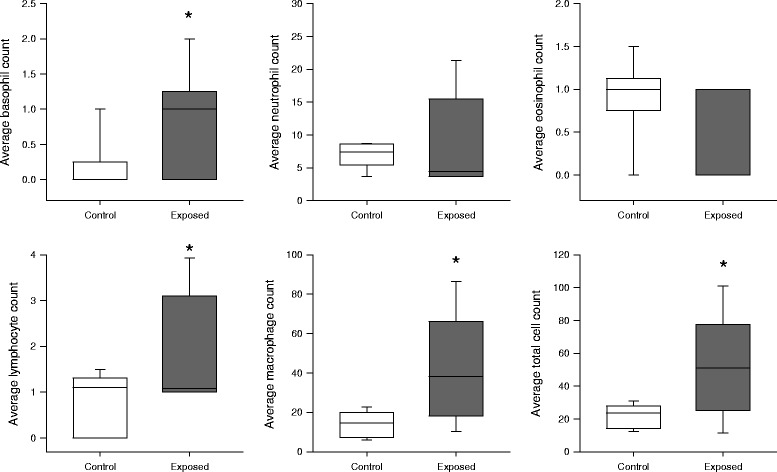
Average number of basophils, neutrophils, eosinophils, lymphocytes, macrophages and total cell counts per field of view in bronchoalveolar lavage fluid samples. *: *p* < 0.05 vs. control group

Total and differential cell counts assessed from BALF are demonstrated in Fig. 4. Samples obtained from the exposed group had elevated numbers of total cell count, macrophages, lymphocytes and basophils (*p* < 0.05 for all) compared to those obtained in the control group. Phagocytized dust particles were observed in 64.9 ± 2 % of the macrophages in the exposed group. Eosinophil and neutrophil numbers exhibited no statistically significant differences.

**Fig. 5:**
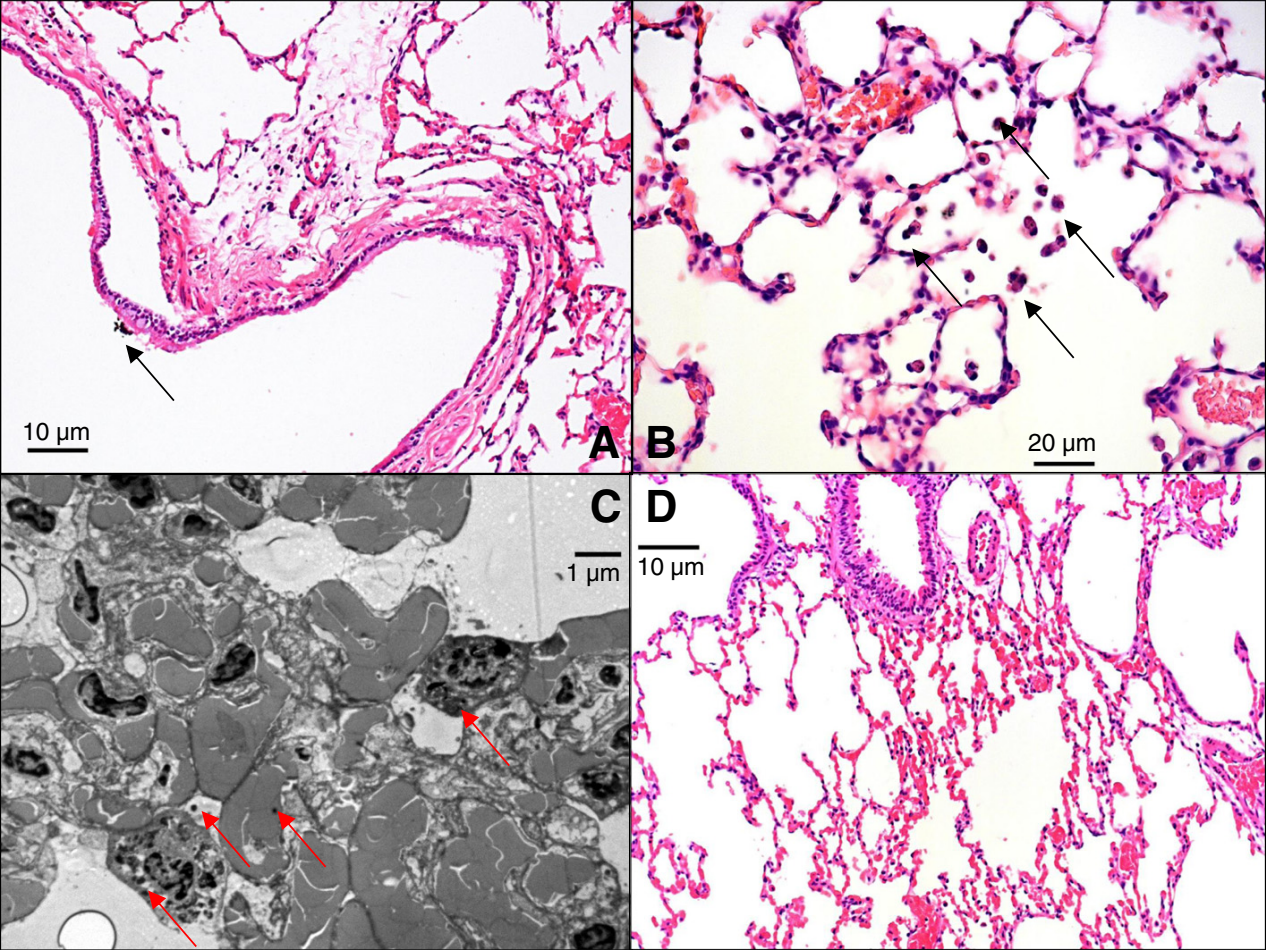
Light (**a**, **b**, **d**) and electron microscopic (**c**) images of the lungs. **a**: Section of a bronchus in a representative animal in the exposed group. Arrow indicates an aggregate of free dust particles inside the bronchial lumen. **b**: Section of the alveolar space in a representative animal in the exposed group. Arrows indicate macrophages with phagocytosed dust particles. **c**: Transmission electron microscopic section of a representative animal in the exposed group. Arrows indicate embedded dust particles. **d**: Alveolar section of a representative animal in the control group

In the light microscopy samples obtained from the animals in the exposed group, free dust particles were observed on the bronchial epithelium (Fig. 5a), and phagocytized dust particles were embedded in the alveolar septa (Fig. 5b). Electron microscopy also revealed the appearance of dust particles in the alveolar macrophages in the animals in the exposed group (Fig. 5c). All these findings were absent in the lungs obtained from the rats in the control group (Fig. 5d).

## Discussion

This study evidenced that a 6-week-long exposure to PM1 at a near-threshold level from a Central European city causes mild airway hyperresponsiveness in healthy adult rats. The respiratory symptoms are not manifested in any adverse changes in the baseline values of the parameters reflecting static lung volume, or airway or respiratory tissue mechanics. However, the presence of mild airway hyperresponsiveness following urban PM1 inhalation suggests the development of airway susceptibilities. To our knowledge, this is the first study to address the pulmonary effects of the continuous inhalation of PM1 at a near-threshold level (concerning to PM10).

### Physical properties and chemical composition of the inhaled PM1

Since the exposure of the rats to PM1 was performed under laboratory conditions, characteristics of the generated aerosol were essential. Mass concentration and particle number size distribution were stable and fulfilled the requirements of the planned protocol (Dp < 1 μm, ρ ~ 100 μg/m^3^) during the exposure periods. As Salma et al. demonstrated by model calculations, the particle diameter applied in this study (391 ± 21 nm) belongs to the most inhalable fraction of the whole size range of atmospheric aerosol [28].

Since the re-suspension of particles was achieved by particle free ambient air, the gaseous composition of inhaled air was identical in case of the exposed and the control animals. Based on the chemical composition of the generated aerosol main emission sources (at the sampling point) were identified. The ratio of BC (indicator of traffic) compared to PM1 mass concentration (BC = 6.38 %) in this study agrees with the findings of other measurements in pedestrian zones in European city centers [29]. Kertész et al. used absolute principal component analysis for source apportionment at the same sampling point as used in this study [30]. According to their results and the identified elements in this study, four sources dominate in the city center of Debrecen: soil (Al, Si, Ca, Fe, Ti), traffic (Cu, Zn, Pb), combustion of oil and coal (S) and a mixed source of power generation and chemical industry (Cl). Since all the identified emission sources are typical of European cities, the findings of this study can be generalized.

### Effects of PM1 on basal respiratory function

To characterize the functional changes in the respiratory system, static lung volume measurements were performed together with an assessment of airway and respiratory tissue mechanics by using the forced oscillation technique. This well-validated technique provides information about the flow resistance of the bronchi (R_aw_) with a detailed description of the respiratory tissue viscoelasticity (G and H). Parameter G reflects the dissipative (damping or resistive) properties of the respiratory tissues, while H is related to the respiratory tissue stiffness (elastance). The baseline values of the EELV [22] and the respiratory mechanical parameters [31, 32] exhibit excellent agreement with those reported previously in rats by using similar experimental methodologies.

Following a 6-week exposure to PM1 at a near-threshold level, no difference was found in the baseline properties of the respiratory system (EELV and mechanical parameters) despite the histological evidence of particles deposited in the acinar and alveolar epithelium. This finding is in concordance with previous results reporting a less than 1 % change in the resistive parameter peak expiratory flow in healthy humans following exposure to diesel exhaust [33], and the lack of change in the forced expiratory lung volumes following exposure to traffic related ambient particles in non-asthmatic subjects [34]. As a mild inflammation of the airways is not associated with a major deterioration of baseline lung function [35], the lack of significant changes in static lung volume, as well as airway and respiratory tissue mechanical parameters is consistent with earlier results, despite the presence of a mild inflammation.

### Airway inflammation and responsiveness following PM1 inhalation

We observed significantly higher increases in R_aw_ and significantly lower PD_50-Raw_ values in rats exposed to nonspecific cholinergic constrictor stimuli, which demonstrated the development of airway hyperresponsiveness. The constriction of the central conducting airways (R_aw_) seems to be unlimited and highly dose-dependent, whereas the lung peripheral response (H) to a cholinergic challenge is restricted. The most plausible explanation for the latter phenomenon may be related to the smaller density of cholinergic receptors [36] on the lung periphery, resulting in their potential saturation by the agonist. The functional abnormality associated with airway hyperresponsiveness was consistent with the development of mild airway inflammation, which was evidenced by the accumulation of macrophages, lymphocytes and basophils in the BALF. We found no evidence for a statistically significant change in the neutrophil cell count in the exposed rats, while exposure to similar nanoparticles led to elevations in neutrophils in earlier studies [37, 38]. This discrepancy can be explained by the larger particle size (391 nm) in our study compared to those ultrafine particles applied previously (25 nm) [38]. Furthermore, the acute phase was investigated in these previous studies, where the innate immunity dominates the inflammatory response, resulting in an elevation of neutrophil count. However, when the exposure is chronic, innate immunity is overpowered by adaptive immunity, resulting in a number of neutrophils around the baseline with elevated lymphocytes.

Histological analyses also confirmed the presence of particles deposited in the bronchial epithelium and phagocytized particles in the alveolar space. Since inflammatory mediators released by these cells were shown to contribute greatly to the development of airway hyperresponsiveness [39], this mechanism provides a plausible explanation to our functional findings. However, the possible involvement of other pathologic processes, such as elevated levels of reactive oxygen species (ROS) and/or oxidative stress can also be anticipated [6, 40, 41].

Due to its technical simplicity, the vast majority of previous studies applied intratracheal instillation of fine and ultrafine particles despite its un-physiological deposition [42]. The few previous studies assessing the respiratory consequences of aerosolized ambient particles demonstrated the appearance of bronchial inflammation [43] and the associated airway hyperresponsiveness [44, 45], similar to our findings. However, these former investigations applied either substantially higher concentrations (3 mg/m^3^) [44], allergen sensitization [43] or short-term (20 min for 7 days) exposure of neonatal subjects [45]. Our findings add to these results the important information that mild airway symptoms may develop at near-threshold concentrations even in a young healthy adult lung.

### Methodological aspects

It must be kept in mind that young healthy adult rats were involved in these investigations. Previous studies report an increased effect of PM in subjects with pre-existing respiratory disorders, such as humans with asthma [34, 46, 47] mice with allergen sensitization [7] or viral infections [37], or in neonatal [45, 48] and aged [38] rat populations.

An important methodological feature of this study is the use of the low-frequency forced oscillation technique to characterize the airway and respiratory tissue mechanics, because it provides the most specific information about the mechanical properties of the different lung compartments. This feature is favorable over methodologies that were applied previously following ambient aerosol exposures, and that supplied either global lung functional indices, such as spirometry [33, 34] or total lung resistance [43, 44], or only qualitative information about the change in the ventilation pattern [14, 35, 37, 43]. However, it is noteworthy that model parameters derived from Z_rs_ data include noticeable components from the chest wall [24, 49]. This suggests that following aerosol exposures the presumably constant chest wall parameters somewhat diminish the real pulmonary changes, particularly in G and H, where the influence of the chest wall is substantial.

## Conclusions

We examined the effects of the 6-week-long inhalation of PM1 from urban aerosol samples on the pulmonary system by performing basal lung function measurements, with the assessment of changes in lung responsiveness and also histopathological analyses. The chemical composition of the generated aerosol was typical of Central European cities, and contained no highly toxic compounds such as heavy metals. Mass concentration was stable during the 6-week-long exposure and never exceeded the current PM10-related alert threshold level by more than 10 %. Following the exposure, hyperresponsiveness and mild airway inflammation were detected in healthy adult rats. Our findings were confirmed by forced oscillatory measurements, cell counts assessed from BALF and histopathological examinations. Former studies of larger particle sizes (PM2.5 or PM10) revealed similar respiratory consequences in case of minimum five times higher mass concentrations. These results suggest that particle size significantly determines the concomitant respiratory responses. Effective prevention could be achieved by taking particle size into consideration when defining air quality standards.

## Abbreviations

BALF: bronchoalveolar lavage fluid
BC: black carbon
BL: baseline
Dp: diameter of particles
EELV: end-expiratory lung volume
G: tissue damping
H: tissue elastance
I_aw_: airway inertance
iv: intravenous
LEZ: low emission zone
MCh: methacholine
NDIR: nondispersive infrared sensor
OPC: optical particle counter
P_1_: pressure at the loudspeaker end of the wavetube
P_2_: pressure at the tracheal end of the wavetube
PAS: photoacoustic spectroscopy
PM: Particulate matter
R_aw_: airway resistance
ROS: reactive oxygen species
TC: total carbon
TEOM: tapered element oscillation microbalance
WD-XRF: wavelength dispersive X-ray fluorescence spectrometry
Z_0_: characteristic impedance of the wave-tube
Z_rs_: input impedance of the respirator system
α: exponent of the constant-phase model
γ: complex propagation number
ρ: mass concentration
ω: angular frequency
